# Synthesis and Biological Evaluation of 2-Picolylamide-Based Diselenides with Non-Bonded Interactions

**DOI:** 10.3390/molecules200610095

**Published:** 2015-06-01

**Authors:** Jamal Rafique, Sumbal Saba, Rômulo Faria Santos Canto, Tiago Elias Allievi Frizon, Waseem Hassan, Emily Pansera Waczuk, Maryam Jan, Davi Fernando Back, João Batista Teixeira Da Rocha, Antonio Luiz Braga

**Affiliations:** 1 Departamento de Química, Universidade Federal de Santa Catarina, UFSC, Florianopolis 88040-900, SC, Brazil; E-Mails: jamal.chm@gmail.com (J.R.); sumbal6s@gmail.com (S.S.); rfscanto@gmail.com (R.F.S.C.); tiagofrizon@gmail.com (T.E.A.F.); 2 Departamento de Bioquímica e Biologia Molecular, Universidade Federal de Santa Maria, UFSM, Santa Maria 97105-900, RS, Brazil; E-Mails: waseem_anw@yahoo.com (W.H.); memypw@yahoo.com.br (E.P.W.); jbtrocha@yahoo.com.br (J.B.T.D.R.); 3 Institute of Chemical Sciences, University of Peshawar, Peshawar 25120, KPK, Pakistan; E-Mail: myemail_id11@yahoo.com; 4 Departamento de Química, Laboratório de Materiais Inorgânicos, Universidade Federal de Santa Maria, UFSM, Santa Maria 97115-900, RS, Brazil; E-Mail: daviback@gmail.com

**Keywords:** diselenides, 2-picolylamide, TPx, TBARS

## Abstract

In this paper, we report the synthesis and biological evaluation of picolylamide-based diselenides with the aim of developing a new series of diselenides with O**···**Se non-bonded interactions. The synthesis of diselenides was performed by a simple and efficient synthetic route. All the products were obtained in good yields and their structures were determined by ^1^H-NMR, ^13^C-NMR and HRMS. All these new compounds showed promising activities when tested in different antioxidant assays. These amides exhibited strong thiol peroxidase-like (TPx) activity. In fact one of the compounds showed 4.66 times higher potential than the classical standard *i.e.*, diphenyl diselenide. The same compound significantly inhibited iron (Fe)-induced thiobarbituric acid reactive species (TBARS) production in rat’s brain homogenate. In addition, the X-ray structure of the most active compound showed non-bonded interaction between the selenium and the oxygen atom that are in close proximity and may be responsible for the increased antioxidant activity. The present study provides evidence about the possible biochemical influence of nonbonding interactions on organochalcogens potency.

## 1. Introduction

Organochalcogen compounds have been explored extensively in many research articles [1,2], reviews [3,4] and books [5,6,7] for their synthetic versatility. Among them, selenium-containing compounds have been successfully employed in certain reactions [8] as catalysts [9], ionic liquids [10], and intermediates in total synthesis [3,4,5,6,7,11]. In this context, the formation of C-Se bonds has contributed to the access to a wide range of biologically active molecules [12,13,14] and functional materials [15].

Synthetic organoselenium compounds have gained special interest since the 1970s, after the identification of various selenoproteins [4,5,13]. These proteins are involved in different physiological processes in mammals, such as antioxidant defense, thyroid hormone production and immune responses [16]. The presence of the selenium atom in such proteins plays a key role in the mode of action, which cannot be performed by sulfur (its closest relative in the chalcogen family) [17]. Due to these findings, a number of new selenium-based pharmaceutical agents are under development for therapeutic use as anticancer, antioxidant, anti-inflammatory and antidepressant drugs, among other applications [18]. Therefore, in pharmaceutical companies and academic laboratories the design and synthesis of biologically relevant small molecules containing selenium constitutes an important research topic.

**Figure 1:**
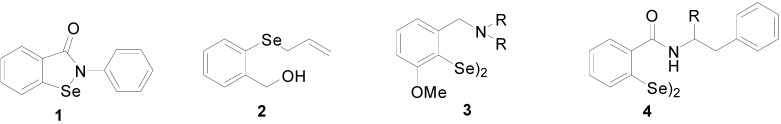
Ebselen (**1**) and organoselenium compounds **2**–**4** with a heteroatom in close proximity.

The literature has shown that in many compounds the selenium atom shows interesting interactions with various heteroatoms like N, O and even S, which lead to possible hypervalent selenium species. This may lead to either intermolecular [19,20] or intramolecular interactions [21,22]. X-ray crystallography [22,23,24], ^77^Se NMR [23,24,25] and modern computational methods [26,27] have been used to explore and provide insights into these possible nonbonding interactions. There are many examples available where organoselenium compounds, e.g., **2**–**3** (Figure 1) [28,29] with proximal interaction showed significant biological activities in comparison to ebselen (**1**; a mimic of GPx that also reacts with peroxynitrite) [5,12].

In this context, our group has recently synthesized diselenides with heteroatoms in close proximity to the selenium atom with interesting GPx-like activity [30,31]. In fact, we have provided conclusive and baseline data that the presence of proximal heteroatom drastically affect GPx-like activity which furthermore enhances both *in vitro* and *in vivo* antioxidant activity with negligible toxicity [32,33,34].

Similarly, 2-picolylamine has been used in the synthesis of many biologically active compounds. For example, there are many reports where picolylamine derivatives were used to form metal complexes as models that mimic both the structure and reactivity of metal ion sites in complex biological systems, showing that picolylamine possess a broad spectrum of biological activities [35,36,37,38].

As part of our wider research program aimed at designing and developing biologically active new organoselenium compounds [15,32,33,34], herein we report a series of 2-picolylamide-based diselenides with proximal non-bonded interactions. The focus of the present paper is to address and explore the synthetic avenues and biosignificance of diselenides with amide groups in close proximity *i.e.*, 2.711 Å. Indeed, pure *in vitro* enzyme mimetic activity of glutathione peroxidase (GPx) and inhibition of iron (Fe II)-induced lipid peroxidation will be assessed in rat’s brain tissue homogenate.

## 2. Results and Discussion

### 2.1. Chemistry

**Scheme 1:**
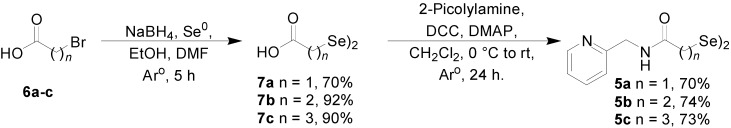
Synthesis of aliphatic diselenide derivatives of 2-picolylamide **5a**–**c**.

We started our studies from the preparation of carboxylic acid diselenide derivatives that were to be used as intermediates to prepare the target compounds. For the synthesis of aliphatic acid diselenides **7a**–**c**, a previously reported method was modified [39,40] starting from the reaction of appropriate bromocarboxylic acids **6a**–**c** with Na_2_Se_2_ (generated *in situ*) as outlined in Scheme 1. The reaction mixture was purified just by solvent extraction, which after vacuum evaporation afforded the pure desired products **7** in good to excellent yields. In the following step, a number of coupling agents were screened to couple carboxylic acid diselenides **7** with 2-picolylamine to form the amide linkage. It was found that DCC and catalytic amount of DMAP constituted the best conditions, resulting in the desired amide **5** in good yields as illustrated in Scheme 1. It is important to mention that when other coupling reagents, such as DIC/HOBt, EDC or BOP were used, the reactions were either not successful or resulted in a complex mixture of byproducts and the starting materials.

**Scheme 2:**
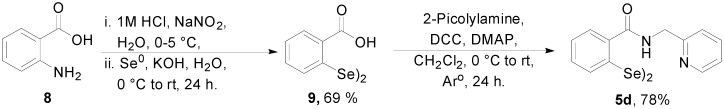
Synthesis of the aromatic 2-picolylamide diselenide derivative **5d**.

The aromatic derivative of amide **5d** was prepared from anthranilic acid (**8**, Scheme 2). Compound **8** was first converted to the bis(2-carboxyphenyl) diselenide **9** through the diazonium salt by treating it with disodium diselenide (generated *in situ*) which on purification by solvent extraction afforded **9** in 69% yield [41]. Subsequently, the diselenide **9** was coupled with 2-picolylamine by the method described previously in Scheme 1.

By applying the strategy shown in Scheme 1 and Scheme 2, 2-picolylamine was easily joined with organoselenium compounds and the biological activities of these diselenides **5a**–**d** were then explored. Furthermore, modification of the distance between amide and selenium through variation of the chain length and the conformation as open or restricted for the interaction between these groups provides some evidence of the influence of the Se-heteroatom interaction between selenium and the amide group on the antioxidant activity of the synthesized molecules.

### 2.2. X-ray Crystallography

**Figure 2:**
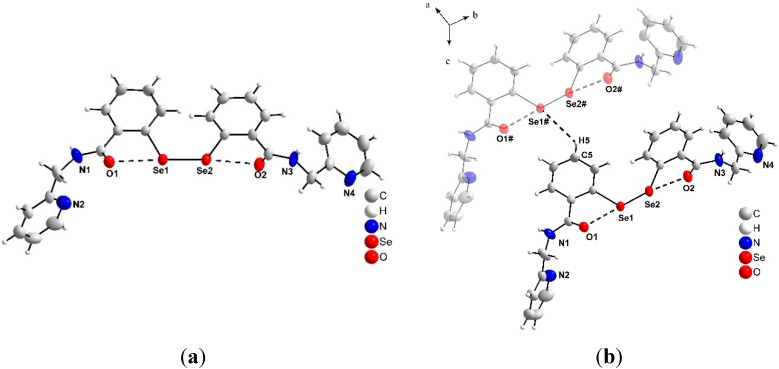
(**a**) Molecular structure of **5d** with 40% thermal ellipsoids (using *DIAMOND* software); (**b**) Molecular and hydrogen interaction assembly of **5d**. Symmetry operations used to generate equivalent atoms: (#) *x*, *y*, −1 + *z*.

Aromatic diselenide **5d** was obtained as solid product, which was crystalized and analysed by the XRD technique. The XRD analysis of **5d** (Figure 2a) shows that the structural parameters of the diselenide are similar to those found in others ligands [42,43]. The Se1-Se2 bond distance [2.3300(3) Å] and the Se-C(Ph) bond distance [1.931(2) and 1.934(2) Å, respective to the Se1-C1 and Se2-C14] are in good agreement with the distances found in other diselenides [44,45].

On the other hand, the Se-O bond distance [2.711(2) and 2.735(2) Å, respective to Se1**···**O1 and Se2**···**O2] is longer than the sum of the covalent radii. However, compared to reports in the literature (O**···**Se 2.793 and 2.852 Å) the distances are in compliance [46], suggesting an intramolecular nonbonding interaction between them. In addition to the intramolecular Se**···**O interactions, some intermolecular interactions between selenium and hydrogen atoms were also found in the crystal lattice. The stark deviation from the interaction of Se2-O2 can be attributed to a weak intermolecular hydrogen interaction between the Se and H (Figure 2b) from the C5 carbon (allowing the visualization of supramolecular assemblies along the two-dimensional plane *bc*.), C5-H5**···**Se1# [H5**···**Se1# = 3.082(1) Å]. The variation that occurs between the distances of Se1-O1 and Se2-O2 atoms provided by hydrogen interactions suggests that on the solid state, there is a difference in reactivity between the selenium atoms. Symmetry transformations was used to generate equivalent atoms: (#) *x*, *y*, −1 + *z*.

### 2.3. Biological Evaluation

Reactive species (RS) play a significant role in normal physiological progressions like, immune function, various signaling cascades and cellular processes (e.g., proliferation, growth, differentiation, apoptosis, migration, cytoskeletal regulation and contraction) *etc.* [47,48,49]. At the same time, RS causes severe damage and injuries to biomolecules like proteins, lipids, lipoproteins and DNA [47,48,49]. As previously discussed, the nonbonding selenium interaction with heteroatoms plays a pivotal role in biological activities. The aim of the present study was to investigate the antioxidant properties of novel chalcogenides. Two different *in vitro* antioxidant assays were performed.

#### 2.3.1. Thiol Peroxidase-Like (TPx) Activity

The catalytic activities of synthesized diselenides **5a**–**d** were examined and the results are summarized in Table 1. Diphenyl diselenide (DPDS), a well-known GPx-mimic, was used as a positive control. The concentrations of new compounds used were 15, 25, 75 and 100 µM. The control or basal GPx-like activity was carried out in the absence of selenium catalysts and the rate of thiophenol oxidation was negligible. Interestingly all compounds exhibited significantly higher catalytic activity.

**Table 1:** Thiol peroxidase like-activity (Δ/Minutes × 1000) of 2-picolylamide-based diselenides **5**.

Compound	Concentration (µM)			
	15	25	75	100
**5a**	1.16	3.88	27.60	34.00
**5b**	0.01	0.01	1.72	3.40
**5c**	9.71	14.97	24.03	31.91
**5d**	20.74	31.34	41.66	46.48
DPDS				9.96

It is apparent from Table 1 that aromatic diselenide **5d** has considerably higher TPx activity (entry 4) than compounds **5a**–**c** (entries 1–3) and DPDS (entry 5). By a closer inspection of the diselenides structures, and specifically **5d**, it is apparent that an adjacent heteroatom, *i.e.*, oxygen, in close proximity of the selenium may possibly generate small nonbonding interactions (between selenium and oxygen). In fact, the experimental data obtained from X-Ray (XRD) analysis of **5d** (Figure 1) confirmed that Se**···**O distance is 2.711 and 2.735 Å, for Se_1_**···**O_1_ and Se_2_**···**O_2_, respectively. Based on interatomic distances (between Se and O1 and O2), we can speculate that Se-O1 may be the initial prime target of the first thiol (PhSH) attacking the Se-Se bond to yield selenyl sulfide.

Using the TPx-like mechanism of the classical standard *i.e.*, DPDS, we can speculate that the Se**···**O interaction in **5d** may promote the conversion of selenyl sulfide to selenol/selenolate. The ease of formation of selenolate ion is a fundamental and significant step. Furthermore, The H_2_O_2_ can oxidize selenolate ion to produce selenenic acid and can regenerate the parent molecule *i.e.*, **5d**, upon reaction with PhSH. It is worthy to note that the presence of adjacent nitrogen (N) in **5d**, may facilitate the attack of incoming thiol (PhSH) on diselenide. The catalytic role of the nitrogen in deprotonation of thiol (PhSH) and consequently a high concentration of thiolate cannot be neglected in this regard [32,50].

In the same context, the literature has demonstrated that the presence of amines in close proximity may possibly interact with selenium and may contribute towards selenolate stabilization and ultimately higher potential [51]. We have recently reported the concise influence of nonbonding nitrogen interaction on the biochemical efficacies of chalcogenides [32,50]. However, it is interesting to note that employing butoxycarbonyl (BOC group), which usually blocks the interactions in synthetic routes, significantly reduced the TPx-like potential of chalcogenides [32]. In fact, the addition of BOC to amino-acid derived diselenides increased the toxic potential apparent from considerably increased ALT activity, serum creatinine and triglyceride levels after *in vivo* treatment in mice [34]. Although not very relevant to selenium molecules, but only to depict the diverse scenarios, we have obtained persuasive evidence that nonbonding interactions can also significantly interact with sulfur (disulfides) [32].

Our results are in strong agreement to above, which may be employed to explain the enhanced TPx potential of **5d** as compared with other analogues **5a**–**c** and the prototypal diselenide DPDS. The TPx-like activity of **5a**–**c** also provides concrete evidence that the length of the carbon chain is important with respect to the interaction between the selenium and the heteroatom (oxygen) in close proximity. Aromatization of this side chain restricts the possible conformations and thus favors the interaction. Based on experimental TPx activity, XRD analysis and relevant literature [32,34,50,51] we can assume the important catalytic influence of nonbonding interactions.

#### 2.3.2. Inhibition of Thiobarbituric Acid Reactive Substances (TBARS) Production in Brain Homogenates

Lipid peroxidation is an important mechanism involved in cellular toxicity. The occurrence of lipid peroxidation specifies the potential disruption of biomembrane integrity and therefore is an important biomarker of oxidative stress. Consequently, the protective effect against lipid peroxidation has an importance for designing new clinically effective antioxidants. TBARS are formed by the reaction of byproducts of lipid peroxidation, mainly malondialdehyde with thiobarbituric acid (TBA). Thus, the stable colored product of TBA with malondialdehyde can be used to quantify lipid peroxidation in biological samples [32,33]. Brain homogenates (S1) can be used as a source of biomembranes and our group has been using this model for some time as an index of lipid peroxidation [32]. During this study Fe(II) was used as an inducer of brain lipid peroxidation as it caused a considerable increase in the TBARS production.

It is worthy to note that Tomoda and Iwaoka method was followed for the determination of TPx-like potential under irrelevant physiological conditions such as using organic solvent (methanol or ethanol) and nonbiological thiols (benzenethiol or thiophenol). At the same time, H_2_O_2_ is used which is a non-reactive oxygen metabolite in the absence of metal ions compared to the hydroxyl radical. Whereas TBARS analysis is performed in complex biological homogenates containing different thiols, metal ions (including iron in different oxidation state), enzymes that can reduce selenium containing molecules (for instance, thioredoxin reductase) lipids, proteins and nucleic acids [52]. Thus, it is rather difficult to do direct comparisons between the GPx- or TPx-like activity and the anti-lipid peroxidation properties of diselenides.

**Figure 3:**
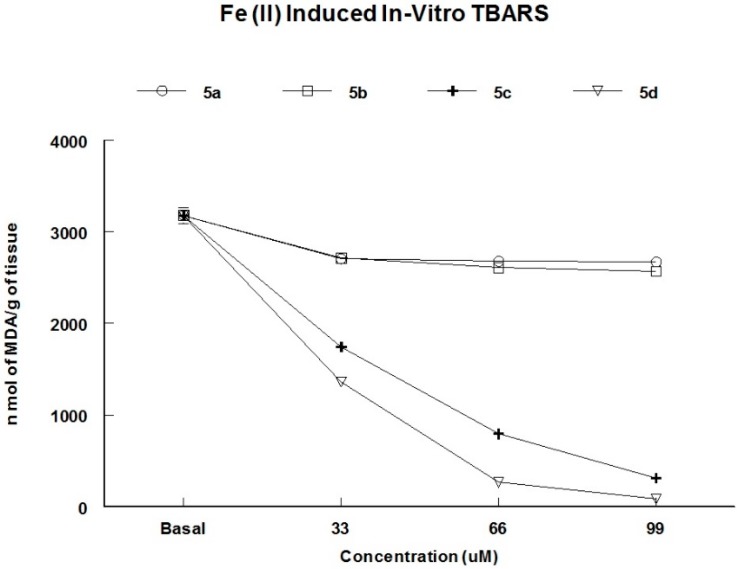
Effect of 2-picolylamide based diselenides **5a**–**d** on Fe (II) induced TBARS formation in low-speed supernatant (S1) from brain homogenate. Fe (II) sulfate at 20 µM was used as pro-oxidant. TBARS are expressed as nmol of MDA/g of tissue. Data are presented as mean ± S.E.M. (*n* = 5).

TBARS assay was performed for the synthesized compounds and the results are outlined in Figure 3. Compounds **5c** and **5d** significantly inhibited TBARS production as compared with **5a**–**b**. The TBARS inhibition potential of **5d** can be attributed to its high TPx-like potential (Table 1). However, compound **5a**, which showed TPx-like activity did not protect lipid peroxidation, and rather **5c** showed interesting protective efficacy in TBARS assay.

## 3. Experimental Section

### 3.1. General Methods and Materials

^1^H- and ^13^C-NMR spectra were obtained at 200/50 MHz on a Bruker AC-200 NMR spectrometer or at 400/100 MHz on a Varian AS-400 NMR spectrometer. Spectra were recorded in CDCl_3_ solutions. Chemical shifts are reported in ppm, referenced to the solvent peak of CDCl_3_ or tetramethylsilane (TMS) as the external reference. Data are reported as follows: Chemical shift (δ), multiplicity, coupling constant (*J*) in Hertz and integrated intensity. Abbreviations to denote the multiplicity of a particular signal are: s (singlet), d (doublet), t (triplet), q (quartet), quint (quintet), sext (sextet) and m (multiplet). The NMR spectra of compounds **5a**–**d** are shown in the Supplementary Material. High-resolution mass spectra were recorded on a Bruker micrOTOF-Q II ESI mass spectrometer equipped with an automatic syringe pump for sample injection. Infrared spectra were recorded on a Bruker Optics Alpha benchtop FT-IR spectrometer and are reported in frequency of absorption (cm^−1^). The melting points were determined in a Microquimica MQRPF-301 digital model equipment with a heating plate. Column chromatography was performed using Silica Gel (230–400 mesh). Thin layer chromatography (TLC) was performed using Merck Silica Gel GF_254_, 0.25 mm thickness. For visualization, TLC plates were either placed under ultraviolet light, or stained with iodine vapor and acidic vanillin. Most reactions were monitored by TLC for disappearance of starting material. All commercially available starting materials were used without further purification. The solvents were distilled and dried according to standard procedures.

Single crystal X-ray diffraction patterns of **5d** were collected with a Bruker APEX II CCD area-detector diffractometer and graphite-monochromatized Mo–*K_α_* radiation. The structure was solved by direct methods using SHELXS [53]. Subsequent Fourier-difference map analyses yielded the positions of the non-hydrogen atoms. Refinements were carried out with the SHELXL package [53]. All refinements were made by full-matrix least-squares on *F*^2^ with anisotropic displacement parameters for all non-hydrogen atoms. Hydrogen atoms were included in the refinement in calculated positions but the atoms (of hydrogens) that are commenting performing special bond were located in the Fourier map. Drawings were done using *DIAMOND* for Windows [54].

#### 3.1.1. General Procedure for the Synthesis of Diselenide **7**

The synthesis was carried out at ambient temperature under inert atmosphere and vigorous stirring. Elemental selenium (200 mesh, 2 g, 0.025 mol) was placed in a two necked round-bottomed flask followed by addition of commercial 80% EtOH (70 mL). NaBH_4_ (1.9 g, 0.05 mol) was then slowly added to the solution, until complete dissolution of selenium and formation of colorless suspension with white-gray solid. Subsequently, DMF (50 mL) was added to the solution and the reaction mixture stirred until the color turned to red-brown followed by addition of commercial 80% EtOH (25 mL). The solution was stirred vigorously until termination of gas evolution and then selenium powder (200 mesh, 2 g, 0.025 mol) was added to the solution and stirred until complete dissolution and formation of clear dark-red solution. The appropriate bromocarboxylic acids **6** (0.05 mol) was slowly added in small portions to the solution, changing the red color of the solution to yellow. After 4 h the reaction was quenched by addition of water (150 mL) and the reaction mixture was extracted with diethyl ether (100 mL) three times. The combined organic phases were washed with water (250 mL) for three times, with brine (100 mL), and was dried over MgSO_4_. The solvent was removed under reduced pressure using rotary evaporator and the crude diselenides **7a**–**c** were used without further purification. Diselenide **7a**: Light yellow solid, 70% yield, mp 101–104 °C (lit. [55] mp 104.5–105.5 °C); **7b**: Yellow solid, 92% yield, mp 132–135 °C (lit. [56] mp 134.5–135.5 °C); **7c**: Yellow solid, 90% yield, mp 86–87 °C (lit. [57] mp 88 °C).

#### 3.1.2. Procedure for the Synthesis of Diselenide **9**

Sodium nitrite (6.9 g, 0.1 mmol,) in water (28 mL) was added dropwise to a stirred solution of anthranilic acid **9** (0.856 g, 6.24 mmol) in 37% hydrochloric acid (20 mL) and water (100 mL) cooled in an ice bath (0–5 °C). The solution of the resulting diazonium salt was stirred for 20 min.

In another flask a mixture of Se powder (16 g, 0.2 mmol) and KOH pellets (24 g, 0.43 mmol) was heated in a round-bottomed flask to 300–360 °C for 2–3 h. The mixture was cooled and distilled water (250 mL) was added. This solution was added dropwise to the about solution of diazonium salt while maintaining the temperature at 0–5 °C which was subsequently, allowed to gain room temperature and left for stirring overnight. The reactions mixture was filtered over Celite. The red solution was acidified with 10% hydrochloric acid forming the precipitate which was collected, resuspended in methanol and refluxed. The suspension was filtered off, and the filtrate was evaporated *in vacuo* yielding the target acid **9** in 69% yield. Diselenide **9**: Yellow solid, mp 297–300 °C (lit. [41] mp 296–297 °C).

#### 3.1.3. General Procedure for the Synthesis of 2-Picolyamide Derivatives of Diselenides **5a**–**d**

The synthesis was carried out at 0 °C under an inert atmosphere under vigorous stirring. The appropriate diselenide (0.005 mol) in dry CH_2_Cl_2_ (5 mL) was placed in a two neck flask followed by addition of a catalytic amount of DMAP (0.366 g, 0.3 mol %). The reaction mixture was stirred for 5–7 min at 0 °C. Next DCC (2.1 g, 0.01 mol, slight excess) was then added to the reaction mixture and further stirred for 15 min at 0 °C. 2-Picolylamine (1.30 mL, 0.010 mol) was added to the reaction mixture and the reaction is left for overnight at ambient temperature. The formation of white precipitate in the reaction was due to dicyclohexyl urea. The reaction mixture was filtered off and then after drying over MgSO_4_, the solvent was evaporated *in vacuo* and the crude product was purified by flash chromatography (ethyl acetate:hexane).

*2,2′-Diselanediylbis(N-(pyridin-2-ylmethyl)acetamide)* (**5a**). Obtained as a yellow viscous liquid in 70% yield; ^1^H-NMR (400 MHz, CDCl_3_) δ: 3.73 (s, 2 H), 4.53 (d, *J* = 5.4 Hz, 2 H), 7.17 (dd, *J* = 7.5, 4.9 Hz, 1 H), 7.30 (d, *J* = 7.9 Hz, 1 H), 7.66–7.61 (m, 1 H), 8.28 (broad s, 1 H), 8.50–8.47 (m, 1 H); ^13^C-NMR (100 MHz, CDCl_3_): 33.0, 45.7, 123.1, 123.2, 137.7, 149.5, 157.6, 171.0; IR (KBr); 3253, 3057, 2926, 2823, 1645, 1560, 1474, 1303, 1215, 758, 528 cm^−1^; ESI-HRMS *m/z*: Calcd. for C_16_H_19_N_4_O_2_Se_2_ [M + H]^+^ 458.9836, found 458.9835.

*3,3′-Diselanediylbis(N-(pyridin-2-ylmethyl)propanamide)*(**5b**). Obtained as a yellow viscous liquid in 74% yield; ^1^H-NMR (200 MHz, CDCl_3_) δ: 2.69 (t, *J* = 7.0 Hz, 2 H), 3.08 (t, *J* = 7.0 Hz, 2 H), 4.49 (d, *J* = 5.1 Hz, 2 H), 7.16 (dd, *J* = 6.9, 5.2 Hz, 1 H), 7.27 (d, *J* = 7.7 Hz, 1 H), 7.63 (td, *J* = 7.7, 1.7 Hz, 2 H), 8.44 (d, *J* = 5.1 Hz, 1 H); ^13^C-NMR (50 MHz, CDCl_3_): 24.3, 37.2, 44.5, 122.4, 122.5, 137.1, 148.7, 156.7, 171.6; IR (KBr); 3291, 3065, 2926, 2823, 1650, 1550, 1435, 1350, 1246, 756, 501 cm^−1^; ESI-HRMS *m/z*: Calcd. for C_18_H_23_N_4_O_2_Se_2_ [M + H]^+^ 487.0149, found 487.0146.

*4,4′-Diselanediylbis(N-(pyridin-2-ylmethyl)butanamide)*(**5c**). Obtained as a yellow viscous liquid in 73% yield; ^1^H-NMR (400 MHz, CDCl_3_) δ: 2.14–2.05 (m, 2 H), 2.39 (t, *J* = 7.3 Hz, 2 H) 2.92 (t, *J* = 7.2 Hz, 2 H), 4.51 (d, *J* = 5.0 Hz, 2 H), 7.17 (dd, *J* = 7.3, 5.2 Hz, 2 H), 7.24 (d, *J* = 7.8 Hz, 1 H), 7.64 (td, *J* = 15.3, 1.7 Hz, 1 H), 8.50 (d, *J* = 5.0 Hz, 1 H); ^13^C-NMR (100 MHz, CDCl_3_): 26.5, 28.7, 35.6, 44.4, 122.1, 122.3, 136.8, 148.9, 156.5, 172.2.; IR (KBr); 3283, 3056, 2926, 1650, 1548, 1435, 1352, 1229, 1176, 756, 511 cm^−1^; ESI-HRMS *m/z*: Calcd. for C_20_H_27_N_4_O_2_Se_2_ [M + H]^+^ 515.0463, found 515.0460.

*2,2′-Diselanediylbis(N-(pyridin-2-ylmethyl)benzamide)*(**5d**). Obtained as a yellow solid in 78% yield; m.p. 138–140 °C; ^1^H-NMR (400 MHz, DMSO-*d_6_*) δ: 4.63 (d, *J* = 5.8 Hz, 2H), 7.30 (dd, *J* = 7.0, 5.8 Hz, 1 H), 7.44–7.35 (m, 3 H), 7.72 (d, *J* = 7.7 Hz, 1 H), 7.80 (td, *J* = 7.7, 1.7 Hz, 1 H), 7.95 (d, *J* = 7.5 Hz, 1 H), 8.55 (d, *J* = 4.7 Hz ,1 H), 9.41 (broad s, 1 H); ^13^C-NMR (100 MHz, DMSO-*d_6_*): 44.5, 120.8, 121.9, 125.9, 127.8, 129.6, 131.6, 131.9, 132.2, 136.7, 148.5, 158.0, 167.2.; IR (KBr); 3324, 3055, 2926, 2851, 1625, 1560, 1474, 1433, 1242, 734, 546 cm^−1^; ESI-HRMS *m/z*: Calcd. for C_26_H_23_N_4_O_2_Se_2_ [M + H]^+^ 583.0151, found 583.0154.

### 3.2. General Methods and Materials

#### 3.2.1. Glutathione-Peroxidase-Like Activity Assay

The catalytic activity of the nitrogen-containing diselenides as a GPx model enzyme was evaluated according to the Tomoda and Iwaoka method [58]. The selenium agent (final concentration: 0.1 mM), thiophenol, PhSH (final concentration 2 mM) at 25(±3) °C were mixed. After 120 s, the catalytic GPx model reaction (H_2_O_2_ + 2PhSH → 2H_2_O + PhSSPh) was initiated by the addition of H_2_O_2_ (final concentration: 5 mM). The reduction of H_2_O_2_ was monitored through the UV absorption increase at 305 nm, due to diphenyl disulfide formation.

#### 3.2.2. Animals

Adult male Wistar rats (250–350 g) from our own breeding colony (Animal House-holding, UFSM, Santa Maria-RS, Brazil) were maintained in room with controlled temperature (22–25 °C), on a 12 h light/dark cycle, with water and food *ad libitum*. Animals were used according to the guidelines of the Committee on Care and Use of Experimental Animal Resources from Federal University of Santa Maria (23081.002435/2007-16).

#### 3.2.3. Thiobarbituric Acid Reactive Substances (TBARS) Assay

Lipid peroxidation was determined by measuring TBARS as described by Ohkawa and coworkers [59] in S1 of brain. Aliquots of S1 (200 μL) were mixed to incubating medium containing Tris-HCl (0.01 mM) pH 7.4, the compounds at different concentrations. Iron (II) as pro-oxidant was added as positive control for lipid peroxidation. The mixture was incubated at 37 °C for 60 min. The basal or pro-oxidant-induced TBARS production was stopped by adding 0.5 mL of acetic acid buffer (pH 3.5), and lipid peroxidation products were measured by the addition of 0.5 mL of TBA 0.6%. Tubes were then incubated in boiling water for 60 min and their contents were subjected to spectrophotometric analysis. The amount of TBARS produced was measured at 532 nm, using MDA as an external standard. TBARS levels were expressed as nmol MDA (malondialdehyde)/g of tissue.

## 4. Conclusions

In conclusion, we have described the synthesis in good yields of new 2-picolylamide-based diselenide derivatives using flexible methodologies. All diselenides were evaluated for their antioxidant capacity to shed light on the SAR on this series of compounds. All these new compounds showed good antioxidant activities when tested in different antioxidant capacity assays. Significantly higher GPx-mimic activity and lipid peroxidation inhibition potential was observed for the synthesized compounds as compared with diphenyl diselenide. In addition, in the present study, crystal structure of the most active compound *i.e.*, **5d**, showed strong nonbonding interaction between the selenium atom and the amide oxygen.

## Supplementary Materials

Additional material available from Cambridge Crystallographic Data Center as Deposition No. CCDC 1061963 comprises H-atom coordinates, thermal parameters and remaining bond lengths and angles. These data can be obtained free of charge via http://www.ccdc.cam.ac.uk/conts/retrieving.html, or from the Cambridge Crystallographic Data Centre, 12 Union Road, Cambridge CB2 1EZ, UK; E-Mail: deposit@ccdc.cam.ac.uk. ^1^H-NMR, ^13^C-NMR, IR and HRMS spectra of **5a**–**d** can be accessed at http://www.mdpi.com/1420-3049/20/06/10095/s1.
