# Supplementary material for: Synthesis and Biological Evaluation of 2-Picolylamide-Based Diselenides with Non-Bonded Interactions
Source: Molecules. 2015 Jun 1;20(6):10095–109. doi: 10.3390/molecules200610095 (PMC6272563; doi:10.3390/molecules200610095)
Supplement: Supplementary file 1 [file molecules-20-10095-s001.pdf]

Supplementary Material

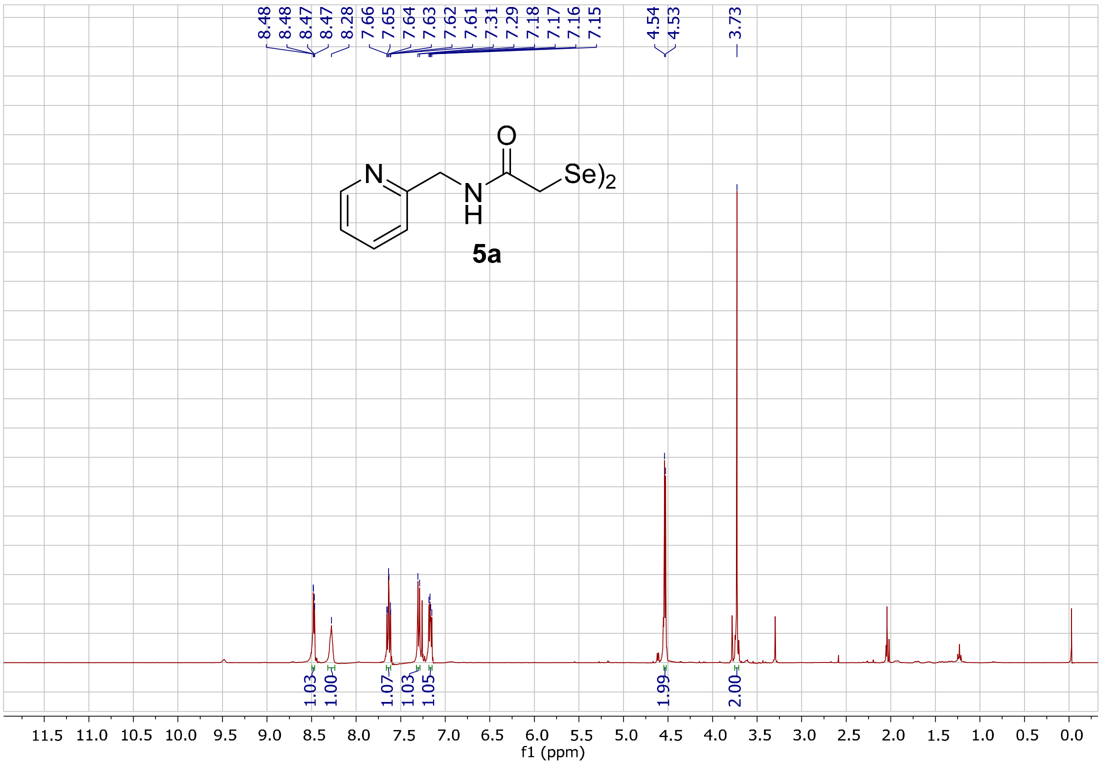

Figure S1. ^{1}H-NMR (400 MHz, $CDCl_3$) spectrum of **5a**.

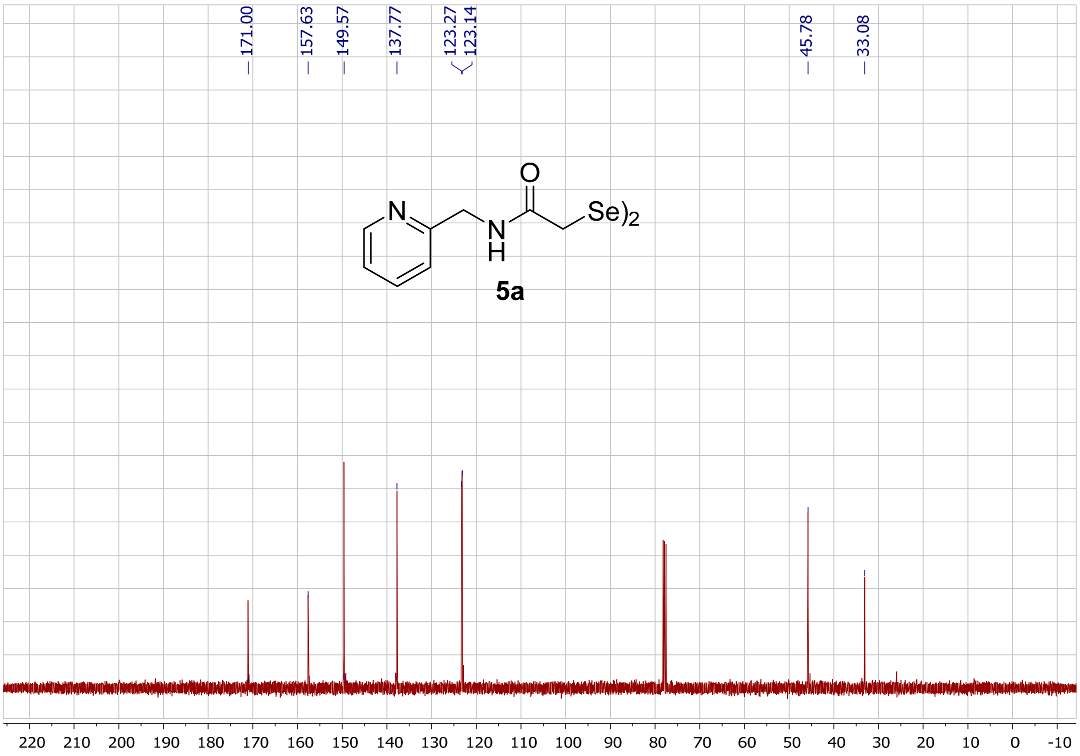

Figure S2. ^{13}C-NMR (100 MHz, $CDCl_3$) spectrum of **5a**.

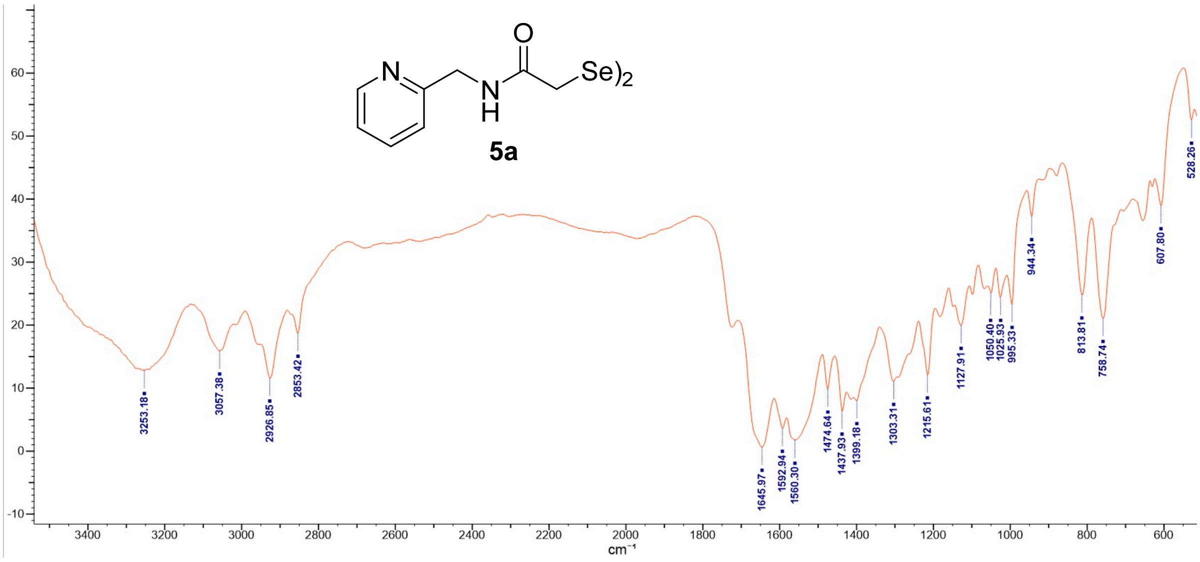

Figure S3. IR spectrum of **5a**.

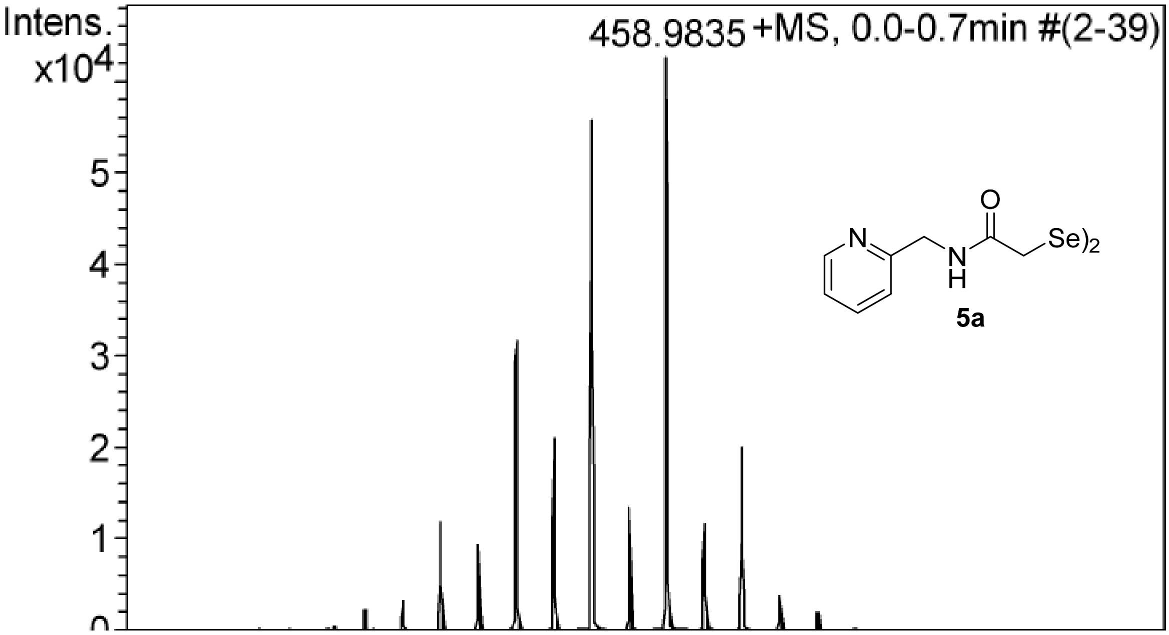

Figure S4. HRMS spectrum of **5a**.

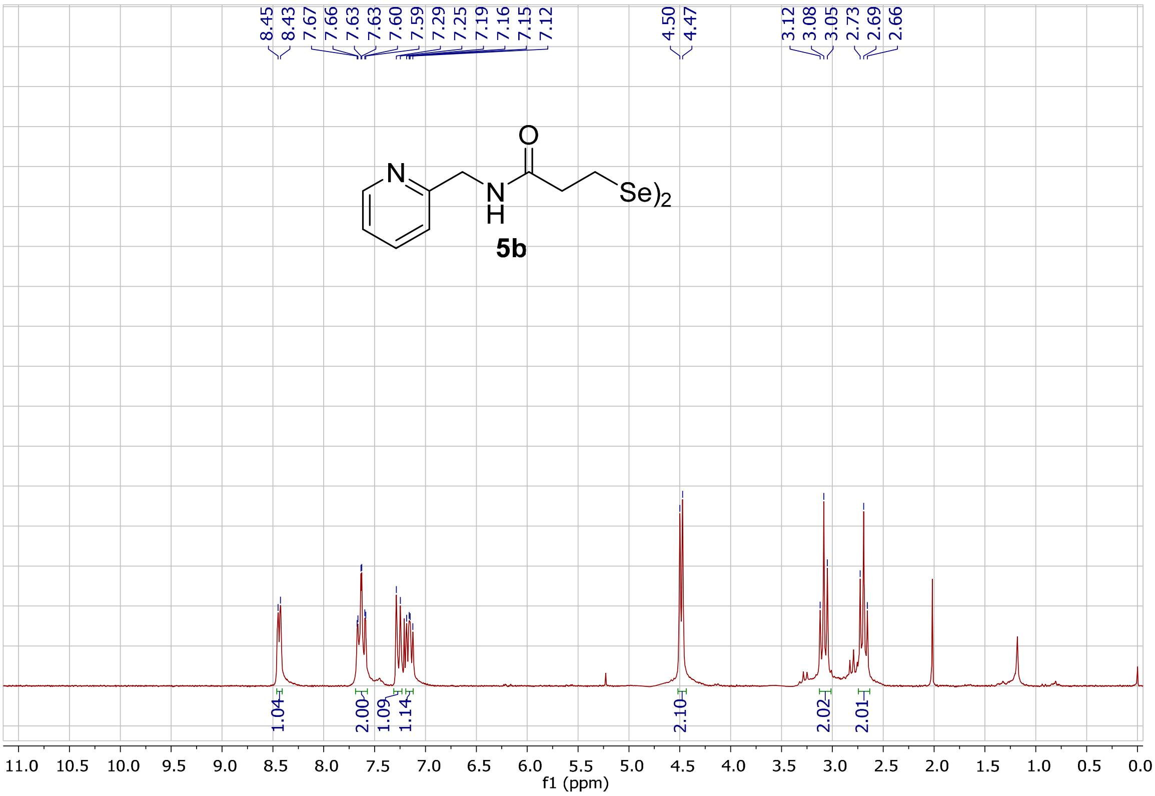

Figure S5. ^{1}H-NMR (200 MHz, $CDCl_3$) spectrum of **5b**.

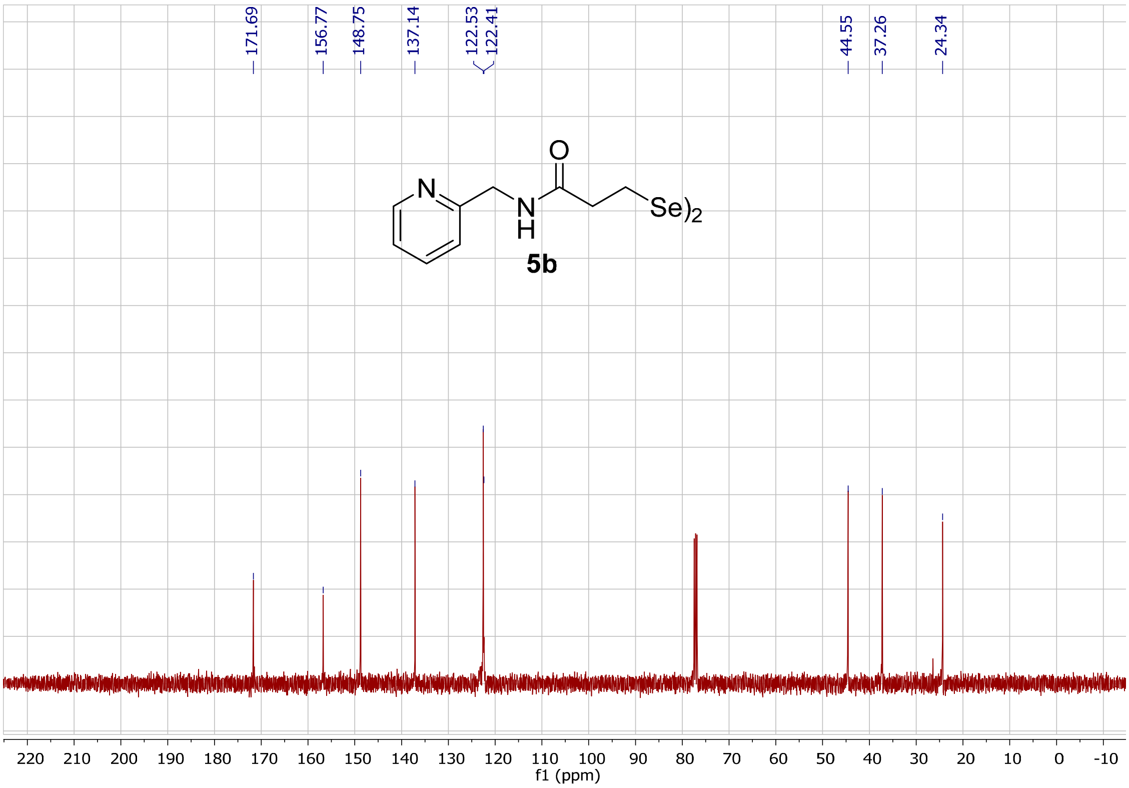

Figure S6. ^{13}C-NMR (100 MHz, $CDCl_3$) spectrum of **5d**.

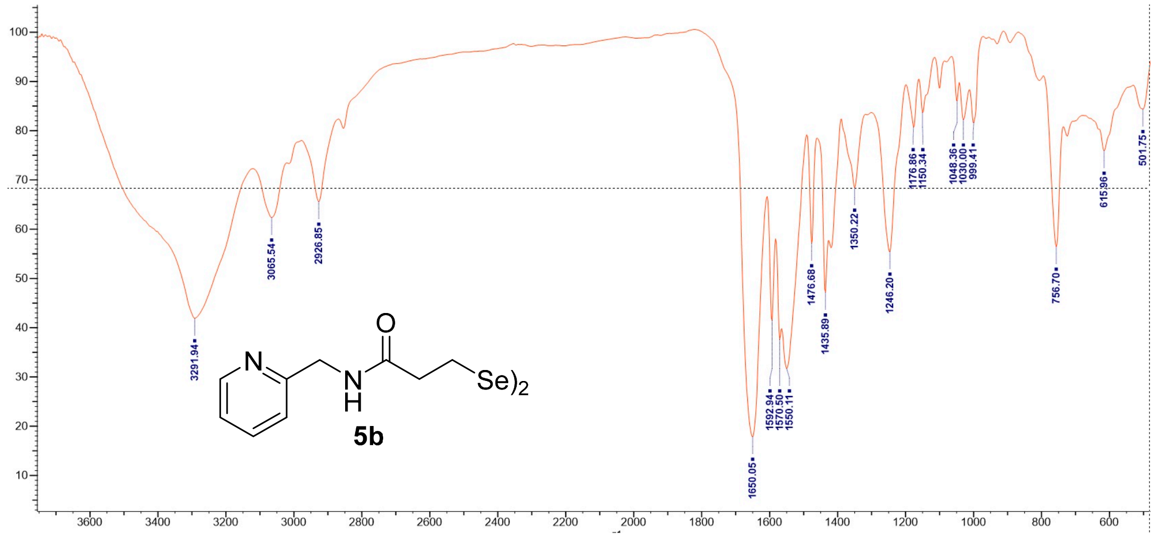

Figure S7. IR spectrum of **5b**.

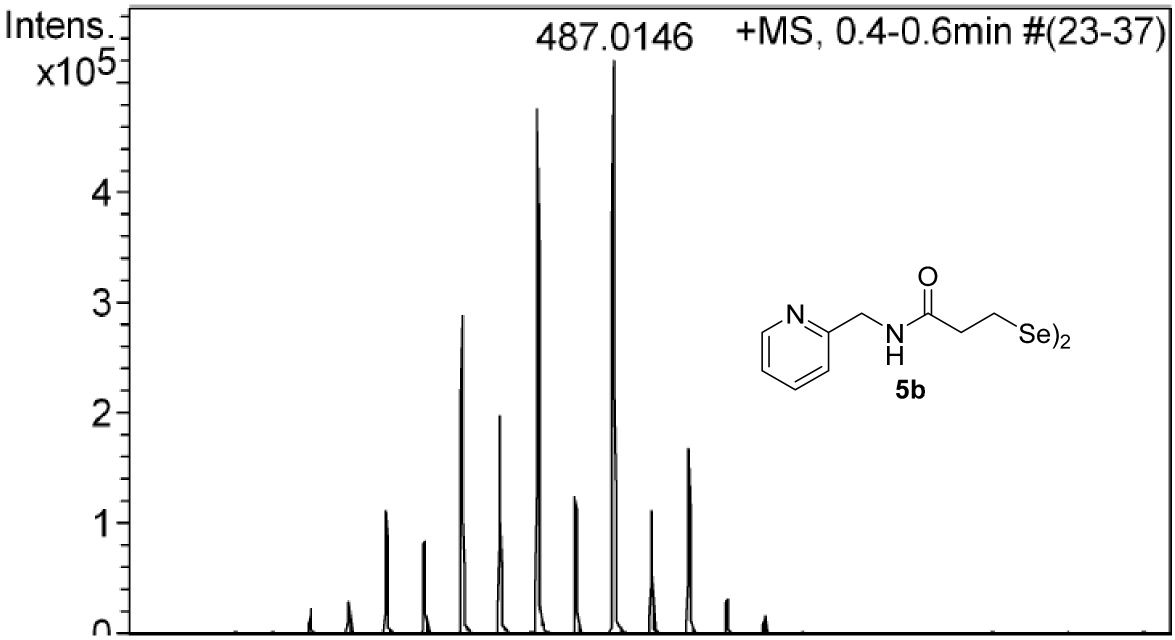

Figure S8. HRMS spectrum of **5b**.

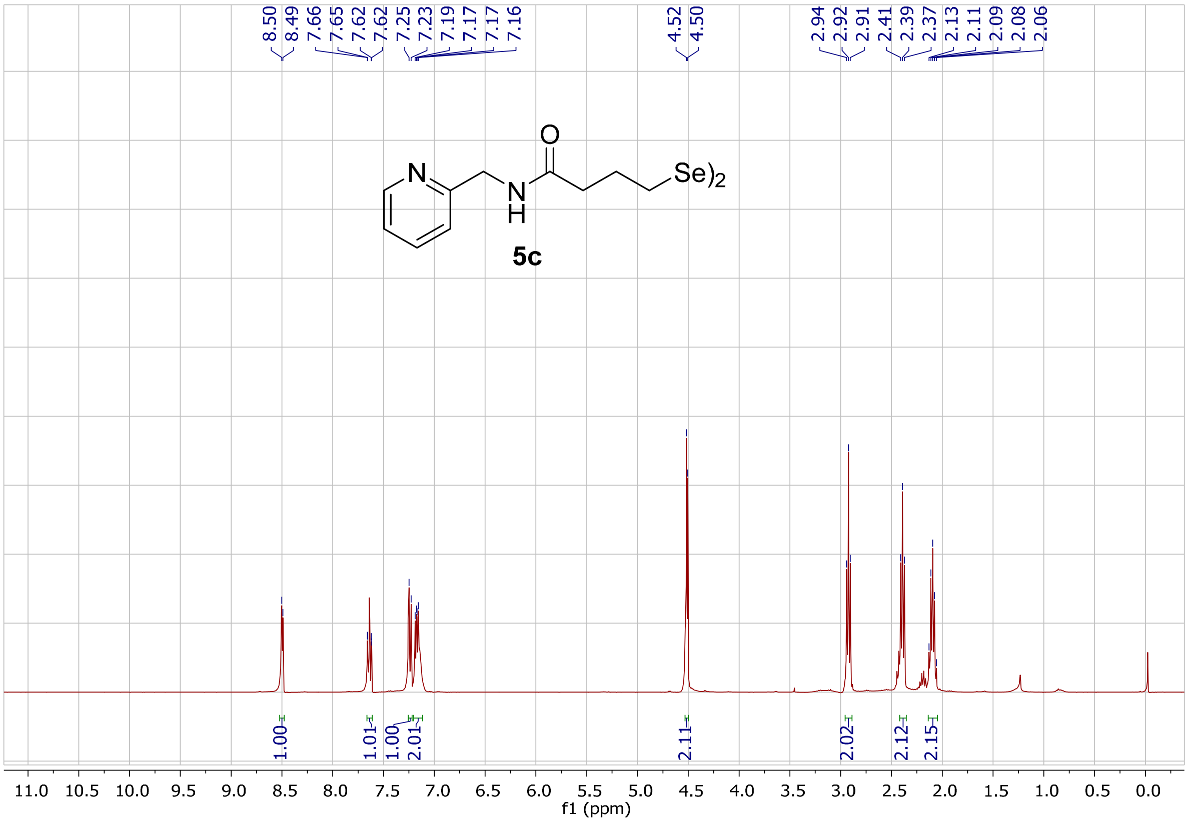

Figure S9. ^{1}H-NMR (400 MHz, $CDCl_3$) spectrum of **5c**.

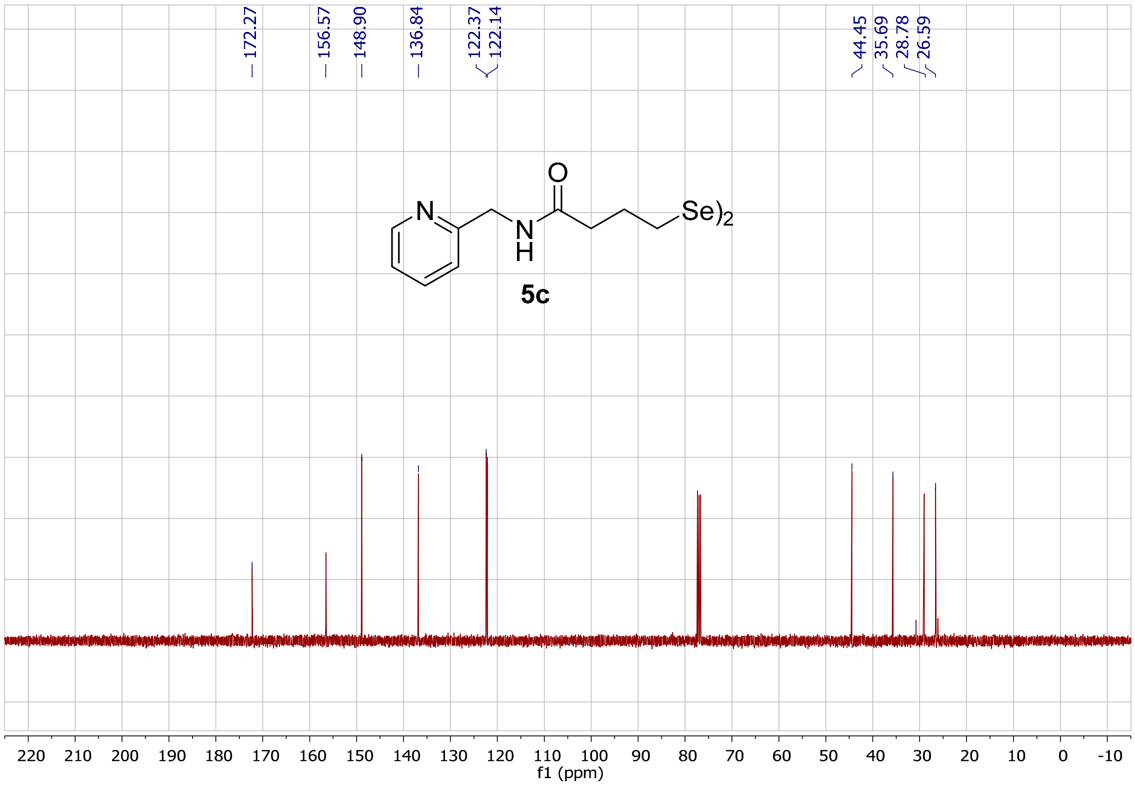

Figure S10. ^{13}C-NMR (100 MHz, $CDCl_3$) spectrum of **5c**.

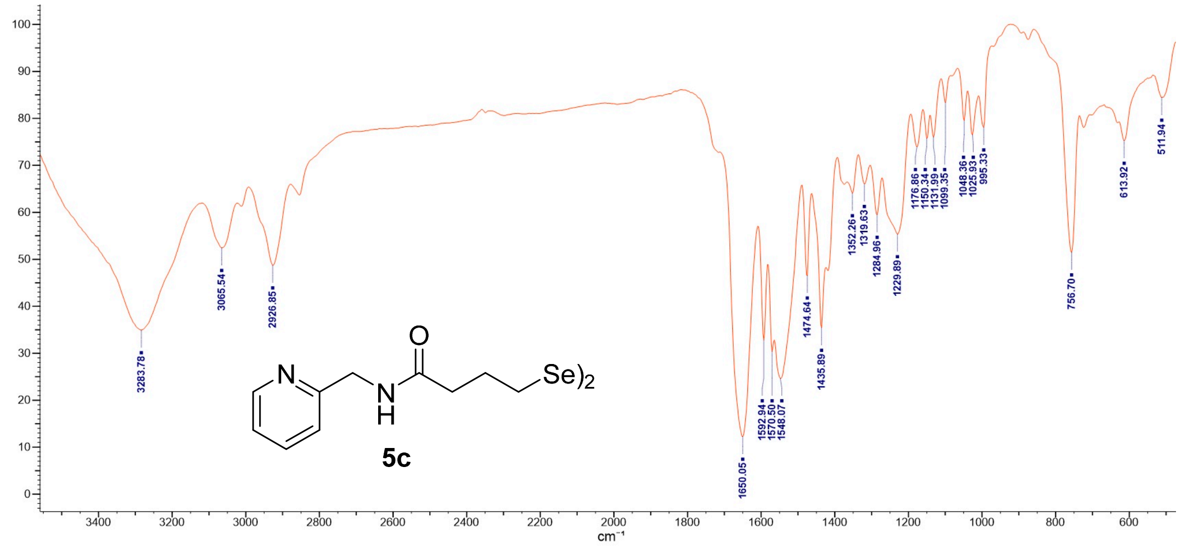

Figure S11. IR spectrum of **5c**.

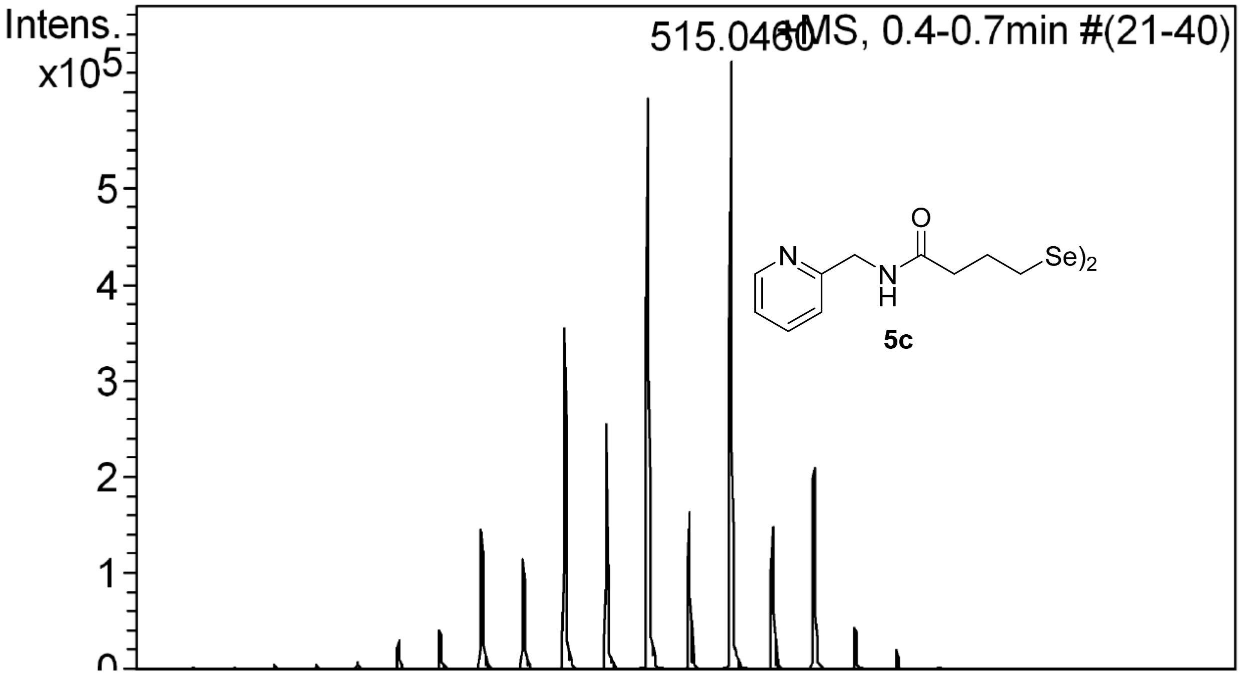

Figure S12. HRMS spectrum of **5c**.

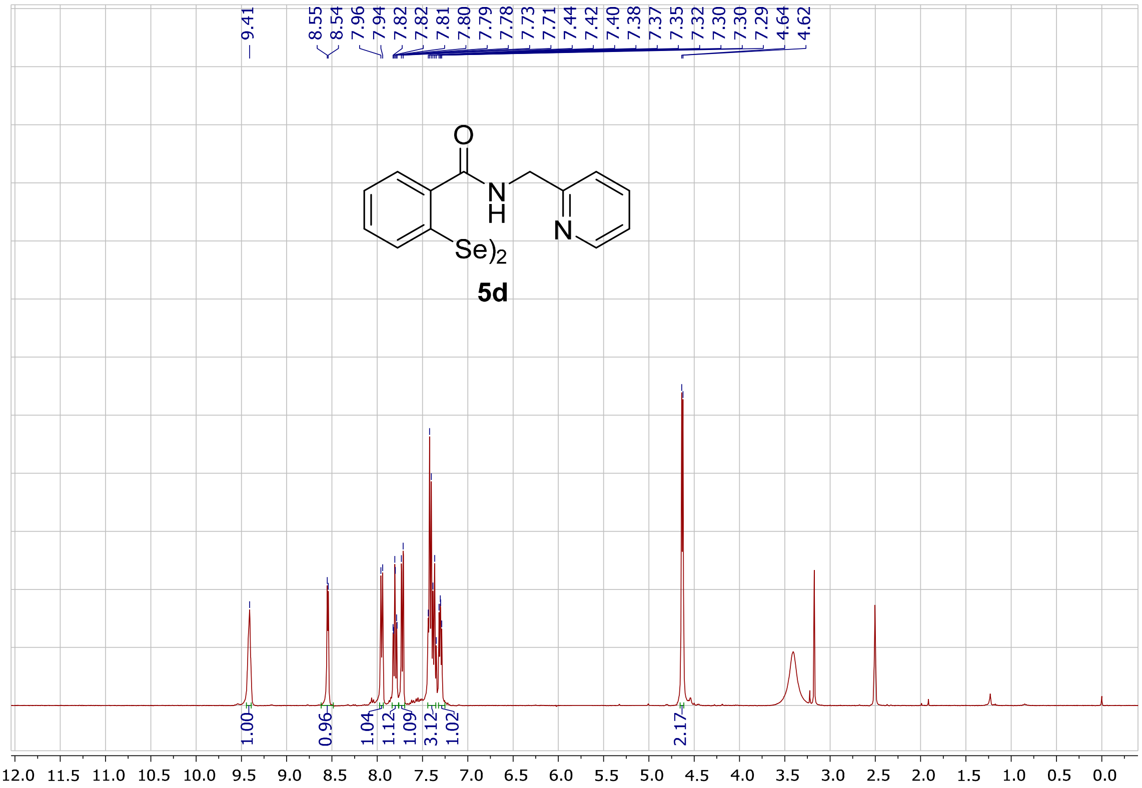

Figure S13. ^{1}H-NMR (400 MHz, DMSO-d_6) spectrum of **5d**.

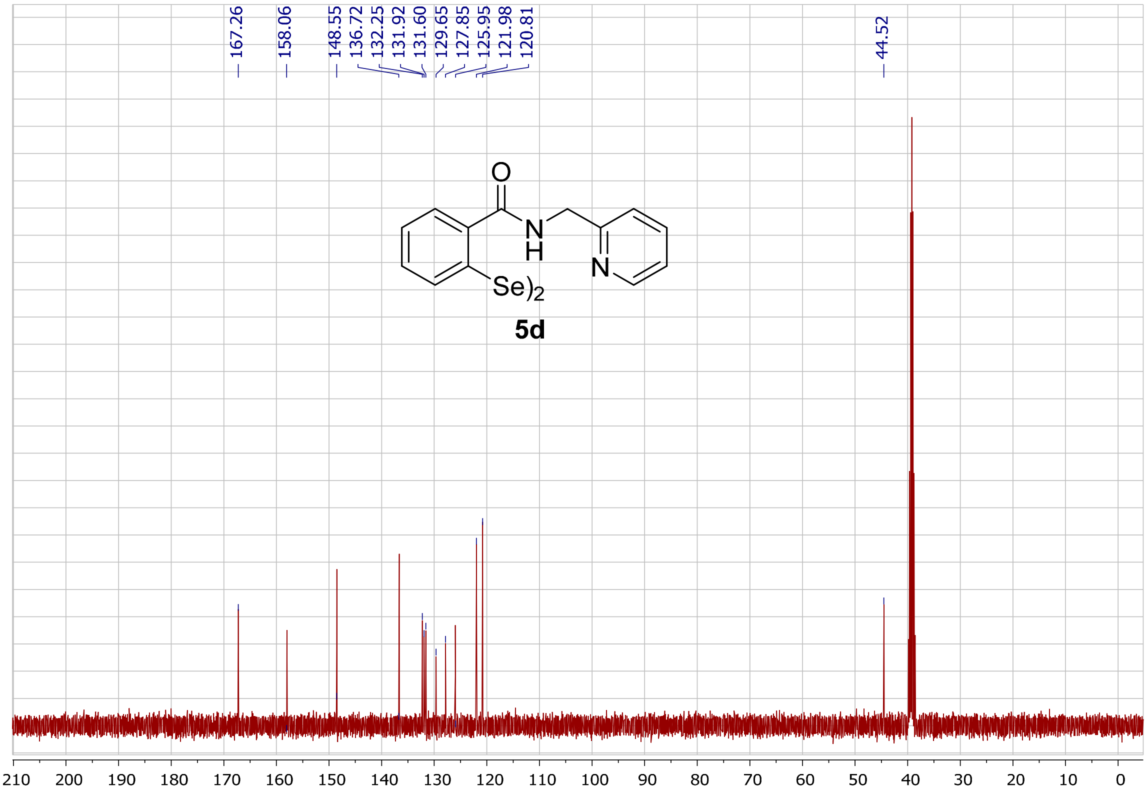

Figure S14. ^{13}C-NMR (100 MHz, DMSO-d_6) spectrum of **5d**.

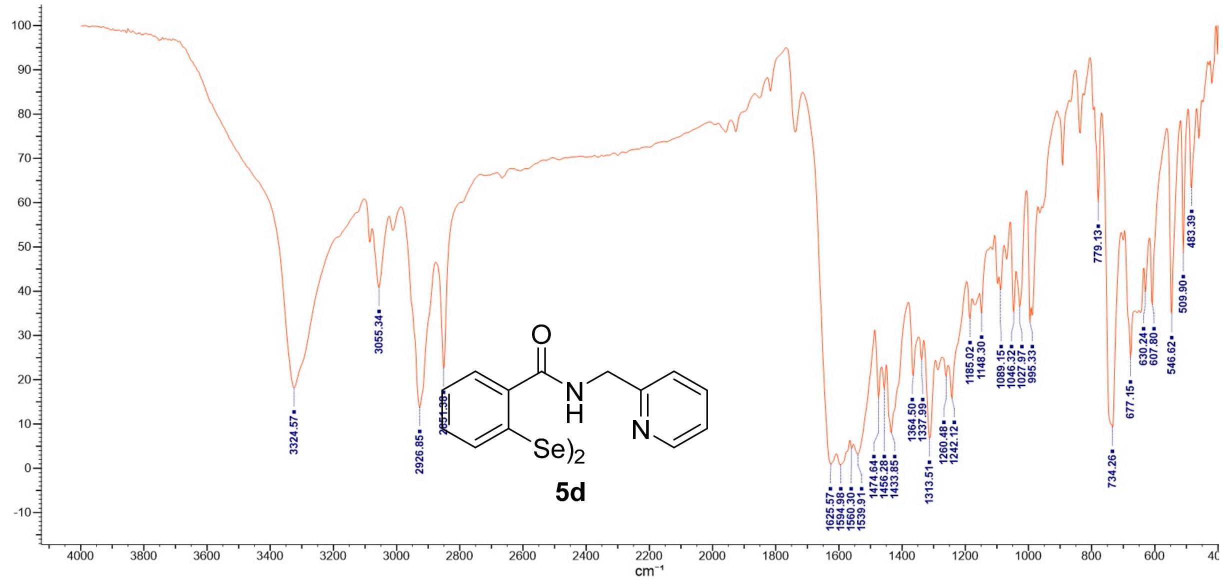

Figure S15. IR spectrum of **5d**.

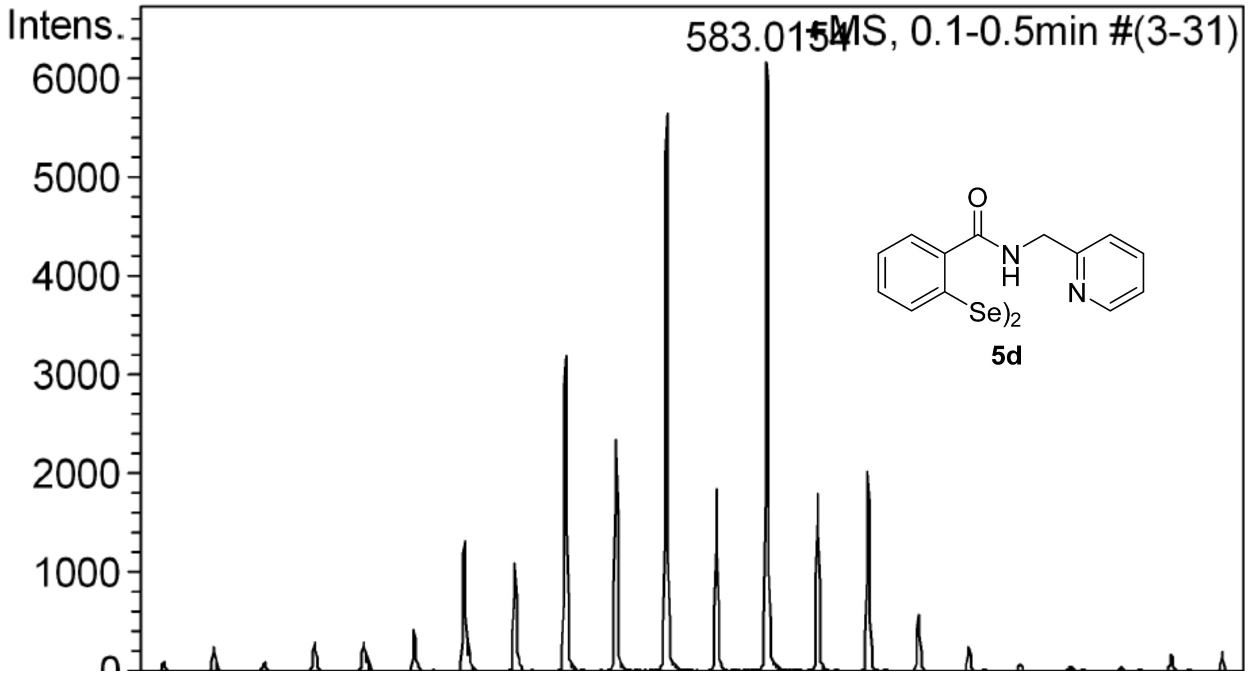

Figure S16. HRMS spectrum of **5d**.

The crystal data and structure refinement for **5d** are summarized in Table S1.

Table S1. Crystal data and structure refinement for **5d**.

Parameters	Value
Empirical formula	$C_{26}H_{22}N_4O_2Se_2$
Formula weight	580.40
Temperature	293(2) K
Wavelength	0.71073 A
Crystal system, space group	Monoclinic, P2(1)
Unit cell dimensions	a = 7.9347(5) Å, α = 90 deg.<br>b = 19.3587(12) Å, β = 109.632(2) deg.<br>c = 8.1377(5) Å, γ = 90 deg.
Volume	1177.33(13) A^3
Z, Calculated density	2, 1.637 $g.m^{-3}$
Absorption coefficient	3.173 mm^{-1}
F(000)	580
Crystal size	0.543 × 0.459 × 0.188 mm
Theta range for data collection	2.10 to 27.25 deg.
Limiting indices	$-10 \leq h \leq 10$,<br>$-24 \leq k \leq 24$,<br>$-6 \leq l \leq 10$
Reflections collected/unique	16292/5151 [R(int) = 0.0261]
Completeness to theta	27.25 (99.8%)
Absorption correction	Numerical
Max. and min. transmission	0.559 and 0.159
Refinement method	Full-matrix least-squares on F^2
Data/restraints/parameters	5151/1/307
Goodness-of-fit on F^2	1.018
Final R indices [I > 2sigma(I)]	R1 = 0.0230, w R2 = 0.0516
R indices (all data)	R1 = 0.0284, w R2 = 0.0534
Absolute structure parameter	0.027(6)
Largest diff. peak and hole	0.295 and −0.309 $e.A^{-3}$
